# The Clinical Significance of Interleukin–1 Receptor Antagonist +2018 Polymorphism in Rheumatoid Arthritis

**DOI:** 10.1371/journal.pone.0153752

**Published:** 2016-04-22

**Authors:** Endom Ismail, Omimah Khaled Jaber Nofal, Rajalingham Sakthiswary, Syahrul Sazliyana Shaharir, Radhika Sridharan

**Affiliations:** 1 School of Bioscience & Biotechnology, Faculty of Science & Technology, Universiti Kebangsaan Malaysia, 43600, Bangi, Selangor, Malaysia; 2 Faculty of Medicine, Department of Medicine, Universiti Kebangsaan Malaysia Medical Centre, Jalan Yaacob Latif, Bandar Tun Razak, 56000, Cheras, Kuala Lumpur, Malaysia; 3 Faculty of Medicine, Department of Radiology, Universiti Kebangsaan Malaysia Medical Centre, Jalan Yaacob Latif, Bandar Tun Razak, 56000, Cheras, Kuala Lumpur, Malaysia; Harbin Medical University, CHINA

## Abstract

**Objective:**

Interleukin-1 receptor antagonist (IL-1Ra) acts as an inhibitor of IL-1; which is one of the culprit cytokines in rheumatoid arthritis (RA). Although +2018 polymorphism of IL-1Ra has been implicated in the pathogenesis of RA, its importance remains poorly understood. Hence, the purpose of this study was to determine the clinical significance of interleukin-1 receptor antagonist (IL-1Ra) +2018 polymorphism in RA.

**Methods:**

Polymerase chain reaction (PCR) and sequencing were used to determine the genotypes of the IL-1Ra +2018 for 77 RA patients and 18 healthy controls. All RA patients were assessed for the disease activity score that includes 28 joints (DAS28) and radiographic disease damage based on Modified Sharp Score (MSS).

**Results:**

The frequency of the T/T and C/T genotypes did not differ significantly (p = 0.893) between the RA patients and the controls. The C/T genotype had significantly higher mean disease activity (DAS 28) and disease damage (MSS) scores with p values of 0.017 and 0.004, respectively. Additionally, the ESR (erythrocyte sedimentation rate), CRP (C-reactive protein), the number of swollen and tender joints were higher for the C/T individuals. On multivariate analysis the CRP, swollen joint count and MSS remained significant with the following p values i.e. 0.045, 0.046 and less than 0.05.

**Conclusions:**

C/T genotype of IL-1Ra +2018 prognosticates more aggressive disease in RA.

## Introduction

Rheumatoid arthritis (RA) is a chronic systemic and inflammatory joint disease that leads to bone and cartilage destruction [1]. It is an autoimmune disease with a complex pathogenesis, with strong contribution by environmental and genetic factors [2].

Cytokines with polymorphic gene sequences have received a great deal of research interest in the recent years. One of the candidate cytokine genes is interleukin 1 (IL-1), which is composed of IL-1α, IL-1β and IL-1 receptor antagonist (IL-1Ra). Genes encoding this family are mapped on chromosome 2q14 [3]. Both IL-1α and IL-1β are potent pro-inflammatory mediators, implicated in human joint destruction [4]. In contrast, IL-1Ra is a naturally occurring anti-inflammatory molecule that inhibits the action of IL-1-induced pro-inflammatory activity [5] and therefore has been shown to prevent joint erosion and damage in RA [2].

Variable number of tandem repeats (VNTR) and +2018 polymorphisms of IL-1Ra have been investigated in several inflammatory diseases including rheumatoid arthritis, systemic lupus erythematosus, alopecia areata and ulcerative cholitis [6–8]. +2018 is a single nucleotide polymorphism (SNP), located in exon 2 of IL-1Ra gene at 2018 nucleotide position. The T allele was much more common in healthy human populations, compared to its other form i.e. the C allele [9, 10]. The C allele has been associated with many human diseases [10–12], albeit data from Asian populations in this regard, remains scarce [13, 14]. The exact roles of the T and C alleles of the IL-1Ra gene in RA have not been fully elucidated. This has prompted us to embark on a study to determine the clinical significance of IL-1Ra +2018 polymorphism in Malaysian RA patients.

## Methods

### Study Design

This was a cross-sectional, case-control study involving RA patients who were under follow-up at the Universiti Kebangsaan Malaysia Medical Centre between January 2014 and May 2015. This study was approved by the Ethics Committee of the institution. All subjects gave written consent to participate in this study. We freshly recruited subjects for this study without using the same subjects of our previous study on interleukin 23 in RA [11]. The subjects were assessed for DAS28 (disease activity score based on 28 joints) and Stanford Health Assessment Questionnaire (HAQ) 8-item Disability Index (HAQ-DI). Seventy seven patients and 18 healthy controls voluntarily consented and were tested for +2018 genetic analysis. The hand radiographs of the subjects were scored using the Modified Sharp Score (MSS) by a radiologist who was blinded to the subjects.

### DNA Extraction and Polymerase Chain Reaction

Genomic DNA was isolated from 200μl whole blood samples using *Qiagen QIAamp DNA kit*, following the manufacturer’s instructions. Genomic DNA was amplified by PCR using the respective forward 5'-GCCTCTTAACCATTGTCAGCC-3' and reverse 5'-GCCTTCTGGTATTTGCCCTT-3 primer following these conditions: initial pre-denaturation 95°C 5 minutes; 34 cycle of denaturation 95°C 1 minute, annealing 66°C 1 minute, extension 72°C 1 minute; and final extension 72°C 10 minutes.

### DNA Purification and Sequencing

The PCR product was purified using the *MEGAquick-spin* Purification kit. Purified PCR products were sequenced by *First BASE Laboratories*. Genotype distribution was determined by repeating unit size of PCR products.

### Statistical Analysis

The statistical analyses were performed using the Statistical Package for Social Sciences (SPSS) package for Windows version 21. Continuous variables were expressed as mean ± SD. Differences between genotype groups were analysed using the Fisher’s exact test for the categorical data whereas the independent *t*-test was used for continuous variables. The odds ratio (OR) was determined using binary logistic regression analysis for variables with significant p values (p < 0.05) on univariate analysis.

## Results

### Demographic Data of the Subjects

A total of 77 RA patients and 18 healthy controls were enrolled in this study. Female RA patients outnumbered the males; 69 (89.6%) versus 8 (10.4%). The mean age of the subjects with RA was 58.6 ± 10.3 years whereas for the controls was 36.4 ± 7.3 years. The demographic characteristics were matched between the RA patients and the controls (Table 1). Our study subjects were mainly of Malay, Chinese and Indian ethnicities reflecting the multiracial Malaysian population. There was a single RA patient who belonged to the Kadazan ethnic group which was categorised as ‘others’. Apart from age and gender, the distribution of the ethnic groups was matched between the RA patients and controls (p = 0.237) as ethnic variation can affect the genetic makeup. The frequency of the T/T and the C/T genotypes did not differ significantly (p = 0.893) between the RA patients and the controls. There was likewise no appreciable difference in the distribution of the allele frequencies.

**Table 1:** Sociodemographic data of the RA patients and the Controls.

	RA patients (n = 77)	Controls (n = 18)	p-value
**Age (years)**	58.6 ± 10.4	36.4 ± 7.3	0.342
**Gender, n(%)**			0.542
**Male**	8 (10.4%)	1 (5.5%)	
**Female**	69 (89.6%)	17 (94.4%)	
**Ethnicity, n(%)**			0.237
**Malay**	42 (54.5%)	16 (88.9%)	
**Chinese**	20 (25.9%)	1 (5.5%)	
**Indian**	14 (18.2%)	1 (5.5%)	
**Others**	1 (1.2%)	0 (0%)	
**IL-1 receptor antagonist genotype, n(%)**			0.893
**T/T genotype**	61 (79.2%)	14 (78.0%)	
**C/T genotype**	16 (20.8%)	4 (22.0%)	
**IL-1 receptor antagonist alleles, n(%)**			0.899
**C allele**	16 (10.39%)	4 (11.11%)	
**T allele**	138 (89.61%)	32 (88.89%)	

Data presented as either counts (percentages) or mean ± SD

### Comparison between the T/T and C/T Genotypes in RA

The C/T genotype had significantly higher mean disease activity (DAS 28) and disease damage (MSS) scores with p values of 0.017 and 0.004, respectively. Besides, the ESR(erythrocyte sedimentation rate), CRP (C-reactive protein), the number of swollen and tender joints were higher in the C/T genotype arm. The differences in the CRP and swollen joint count was statistically significant (p<0.050 and 0.033, respectively) (Table 2). The above differences were observed despite the matched frequency of seropositivity, disease duration and usage of medications between both the genotypes. On multivariate analysis (Table 3), the CRP, swollen joint count and MSS remained significant with the following p values: 0.045,0.046 and <0.05.

**Table 2:** Clinical Characteristics of the RA patients.

Parameters	IL-1 Receptor Antagonist		p value
	T/T genotype	C/T genotype	
Disease duration (years)	10.30 ± 7.70	10.10 ± 9.90	0.747
Positive RF, n(%)	43 (70.5)	12(75.0)	0.299
CRP (mg/dL)	0.53 ± 0.67	1.30 ± 2.02	**<0.050**
ESR (IU/ml)	44.65 ± 23.40	48.00 ± 27.30	0.757
HAQ DI HAQ < 1, n(%)	59 (96.7)	15 (93.7)	0.138
HAQ ≥ 1, n(%)	2 (3.3)	1 (6.3)	
MSS	4.22 ± 8.74	24.44 ± 10.71	**0.004**
Swollen joint count	0.92 ± 1.92	1.69 ± 0.95	**0.033**
Tender joint count	0.69 ± 1.46	1.19 ± 1.17	0.907
VAS	3.58±2.12	3.06±1.84	0.446
DAS 28 scores	2.03±1.15	2.79±0.89	**0.017**
Disease Activity			
Remission–low, n(%)	52 (85)	11 (64.5)	0.183
Moderate–high, n(%)	9 (14.7)	5 (35.5)	** **
Medications, n(%)			0.597
Methotrexate	44 (72.00)	11 (68.80)	
Leflunomide	23 (37.70)	3(18.75)	
Sulfasalazine	14 (23.00)	7 (43.75)	
Hydroxychloroquine	6 (9.80)	1 (6.25)	
Prednisolone	1 (1.60)	4 (25.00)	
Biologics	3 (4.90)	1(6.25)	

CRP; C reactive protein, ESR: erythrocyte sedimentation rate, HAQ-DI; Health Assessment Questionnaire Disability Index, MSS: Modified Sharp Score, VAS: visual Analog Score, DAS 28: 28 joint based Disease Activity Score

Data presented as either counts (percentages) or mean ± SD

**Table 3:** Multivariate Analysis.

Variable	Odds ratio	p value	95% CI
DAS 28	2.206	0.170	0.712–6.838
CRP	3.925	**0.045**	1.030–14.960
Swollen Joints	0.399	**0.046**	0.162–0.985
MSS	1.322	**<0.05**	1.142–1.532

CRP; C reactive protein, MSS: Modified Sharp Score, DAS 28: 28- joint based Disease Activity Score

## Discussion

Advances in genetic studies have lead to the discovery of a wide variety of genetic loci which may contribute to the development and course of RA [12]. HLA alleles, particularly HLA-DRB1 shared epitope alleles, have been shown to dominate the genetic risk in RA, while other less predominant loci includes the non-HLA variants [13] and multiple single nucleotide polymorphisms (SNPs) such as *VEGFA* [14], *RANKL[15]*, *MMP1-3* [14] and *PTPN22* [16].

IL-1 signalling pathway is a critical cytokine pathway in RA which is involved in cell migration and inflammation [17]. The regulation and secretion of the cytokine has been demonstrated to be under genetic control through genetic polymorphism in their coding and promoter gene sequences [18]. Therefore, IL-1Ra polymorphism, has been postulated to have a pivotal role in RA [19–22]. However, the data is still inconclusive owing to the conflicting findings of many of the studies in this regard [23].

We found no significant difference in the frequency of the IL-Ra C/T and T/T genotype expression between the RA patients and the controls. In keeping with our findings, Arman et al [24] revealed that their 94 RA patients and the 104 healthy controls had similar +2018 genotype distribution. The same finding was reported by another Asian study by Lee et al [10].

The novel findings of our study include the associations of the C/T genotype of IL-1Ra+2018 with significantly higher disease activity based on DAS28 scores and disease damage based on MSS scores. A few studies have consistently pointed out the role of IL-Ra gene polymorphism in the disease activity of RA. For instance, RA patients with persistent active disease, had significantly increased expression of IL-1RN*1 and IL-1RN*2 [25]. Apart from that, another study which involved Spanish RA patients also found a significant association between homozygosity for IL1RN*2 with increased number of affected joints [20]. In parallel with our findings, an Italian study demonstrated that their RA patients with C/T genotype of IL-1Ra had more aggressive disease with poor response to methotrexate therapy [22]. Polymorphisms in the regions of cytokine genes that affect transcription may influence cytokine production [26]. It is tempting to speculate that the higher disease activity among RA patients with C/T genotype of +2018 was due to increased production of IL-1 by monocytes. Unfortunately, data on the production of cytokines in relation to single chromosomal markers in the IL-1 locus are prone to experimental errors and hence, remain controversial.

Unlike disease activity, not many studies in the past have disclosed a link between IL-1Ra genotypes and the disease damage in RA. Recent studies by Gaafar et al [27], Cantagrel et al [28] and Buchs et al [29], for instance, failed to demonstrate that IL-1Ra polymorphism was predictive of more severe joint destruction. Kazkaz et al [30] found that tumour necrosis factor alpha promoter -238 but not IL-Ra +2018; was associated with joint destruction based on Larsen scores of the right wrist. However, Lubbe et al [31] disclosed that the +2018 “C”allele predicted higher Larsen scores which was consistent with our findings of higher disease damage with C/T genotype. Cox et al.[32] found 4 IL-1 gene cluster markers which were associated with joint erosions. It is plausible that the more severe joint destruction is due to the stimulation of bone resorption by IL-1 due to genetic linkage disequilibrium [33]

In conclusion, C/T genotype of +2018 prognosticates more aggressive disease in RA. The above genotype is associated with higher disease activity and more extensive radiographic joint damage. These findings, albeit interesting, have to be considered preliminary and will need to be confirmed by future genetic studies. However, owing to the polygenic aetiology, ethnic variations and complexity of the disease itself, there may be lack of reproducibility of genetic associations in RA.

## Supporting Information

S1 Appendix Raw data. (XLSX)
